# An android-smartphone application for rice panicle detection and rice growth stage recognition using a lightweight YOLO network

**DOI:** 10.3389/fpls.2025.1561632

**Published:** 2025-04-16

**Authors:** Huiwen Zheng, Changjiang Liu, Lei Zhong, Jie Wang, Junming Huang, Fang Lin, Xu Ma, Suiyan Tan

**Affiliations:** ^1^ College of Electronic Engineering, South China Agricultural University, Guangzhou, Guangdong, China; ^2^ College of Mechanical and Electrical Engineering, Xinjiang Agricultural University, Urumqi, China; ^3^ College of Engineering, South China Agricultural University, Guangzhou, Guangdong, China

**Keywords:** rice panicle, growth stages, YOLOv8, lightweight YOLOv8, android application

## Abstract

**Introduction:**

Detection of rice panicles and recognition of rice growth stages can significantly improve precision field management, which is crucial for maximizing grain yield. This study explores the use of deep learning on mobile phones as a platform for rice phenotype applications.

**Methods:**

An improved YOLOv8 model, named YOLO_Efficient Computation Optimization (YOLO_ECO), was proposed to detect rice panicles at the booting, heading, and filling stages, and to recognize growth stages. YOLO_ECO introduced key improvements, including the C2f-FasterBlock-Effective Multi-scale Attention (C2f-Faster-EMA) replacing the original C2f module in the backbone, adoption of Slim Neck to reduce neck complexity, and the use of a Lightweight Shared Convolutional Detection (LSCD) head to enhance efficiency. An Android application, YOLO-RPD, was developed to facilitate rice phenotype detection in complex field environments.

**Results and discussion:**

The performance impact of YOLO-RPD using models with different backbone networks, quantitative models, and input image sizes was analyzed. Experimental results demonstrated that YOLO_ECO outperformed traditional deep learning models, achieving average precision values of 96.4%, 93.2%, and 81.5% at the booting, heading, and filling stages, respectively. Furthermore, YOLO_ECO exhibited advantages in detecting occlusion and small panicles, while significantly optimizing parameter count, computational demand, and model size. The YOLO_ECO FP32-1280 achieved a mean average precision (mAP) of 90.4%, with 1.8 million parameters and 4.1 billion floating-point operations (FLOPs). The YOLO-RPD application demonstrates the feasibility of deploying deep learning models on mobile devices for precision agriculture, providing rice growers with a practical, lightweight tool for real-time monitoring.

## 1 Introduction

Rice is one of the most important food crops in the world. With the continuous growth of the world’s population, food production has become particularly important, so it is urgent to cultivate high-yield rice to alleviate the problem of food shortages. As the most important phenotype of rice, rice panicles are not only closely related to crop yield but also play an important role in disease detection (Deng et al., 2019), crop organ detection (Zhang et al., 2022), and reproductive stage identification (Ikasari et al., 2017). Identifying the growth stages of rice helps the implementation of appropriate irrigation, fertilization, and pesticide applications during the suitable growth stages, achieving precise field management and ensuring the maximum yield (Gagic et al., 2021). However, at present, the detection of rice panicles and the identification of rice growth stages are mainly based on manual work, which has low efficiency and strong subjectivity. As deep learning has become a prevalent way of target detection and easy-carry smartphones are becoming more powerful tools, employing deep learning models for online detection tasks in an easy-to-use smartphone application tool for rice panicle detection is of great significance and has had promising results.

With the rapid development of image processing technology and the emergence of deep learning methods, many studies have used this technology for crop organ detection. For example, a corn panicle detection algorithm was developed by Brichet et al. (2017) based on the random forest algorithm and Visual Geometry Group 16-layer network (VGG16). They processed 12 whole-plant side-view images obtained from a camera and determined the position of the panicle based on changes in stem width. Hong et al. (2022) proposed an improved Mask R-CNN combined with Otsu preprocessing for rice panicle detection and segmentation. This method achieves good detection and segmentation accuracy for rice grains and performs well in large-scale field environments, making it suitable for rice growth detection and yield estimation. Zhao et al. (2022) proposed a wheat-oriented spike detection method called OSWSDet based on deep learning. They processed images of wheat fields collected by drones and used a circular smooth integration method, CSL, and a micro-scale detection layer combined with the YOLO framework to detect wheat spikes. The results showed that the OSWSDet method was superior to traditional wheat spike detection methods, with an average accuracy of 90.5%. Dandrifosse et al. (2022) used an unsupervised learning method based on YOLOv5 and the DeepMAC segmentation method to count and segment wheat RGB images from the heading stage to maturity. Only a small amount of labeling work is required to start training. The average F1 score for wheat spike detection was 0.93, and the average F1 score for segmentation was 0.86.

In addition, deep learning is widely used to recognise the crop growth stage. Yang et al. (2020) used mono-temporal unmanned aerial vehicle (UAV) imagery to estimate crop phenology after network training and used convolutional neural networks (CNNs) that combined with Spatial Pyramid Pools (SPPs) to identify key phenological periods of rice, which proved the effectiveness of CNN technology for near-real-time phenology detection of rice and harvest time estimation. Bai et al. (2018) transformed the observation of the rice heading stage into the detection of the rice spike and proposed a new method to automatically observe the rice heading stage using SVM with color features and a gradient histogram as the input and a CNN. The results showed that the time difference between this method and manual observation was within 2 days, and it could replace manual observation. Zhang et al. (2022) used an improved convolutional neural network to detect rice panicles. Inception ResNet-v2 was used to replace VGG16 as the feature extraction network, a feature pyramid network (FPN) was used to integrate with a region proposal network (RPN), and non-maximum suppression (NMS) adopted the DIoU standard. The average accuracy of rice panicle detection was 92.47%. The growth stage of rice was determined by rice panicle density, and the deviation between the result and manual observation was within 2 days, which could meet the needs of agricultural activities. Conventionally, the rice growth stage is determined by the number of panicles or panicle density. However, there are significant changes in the external morphological structure of rice panicles in several key growth stages, such as shape, color, size, texture, and posture. This allows us to explore and observe rice panicles, detect them, and then recognize different key growth stages in rice.

In terms of support equipment, target detection based on deep learning models is mostly performed on computer devices, which are very inconvenient for small-scale farms. However, with advantages in terms of computing power, low price, ease of use, and portability, smartphones have become a prevalent tool for target detection and have achieved promising results. Numerous smartphone applications have been reported in agricultural automation, such as fruit detection, disease detection (Anbarasi et al., 2019), and weed identification. In addition to these studies, there have also been reports on crop phenotyping automation using smartphone applications. Komyshev et al. (2017) adopted a method for the automated assessment of wheat phenotypic parameters using an Android mobile device, and the experimental results showed that the method was able to efficiently and accurately assess the phenotypic characteristics in wheat grains. The evaluation of the application under six different illumination conditions and on three mobile devices showed that the illumination conditions had a significant effect on the accuracy. Zeng et al. (2023) presented a lightweight YOLOv5-based algorithm for real-time tomato detection. It incorporates techniques such as replacing the focus layer with a downsampling convolutional layer, using the MobileNetV3 bneck module, and applying channel pruning. The model achieved a 78% reduction in parameters and model quantization for mobile devices increased the frame rate by 268%, while maintaining a 93% true detection rate. Tao et al. (2020) developed a smartphone app based on a standard leaf color chart (LCC) to detect the color levels of rice leaves using color threshold segmentation. A CIELAB histogram was used to extract the color features of each region, and the CIEDE2000 formula was used to distinguish the color grade of the rice leaves. The accuracy of the app in determining the color levels of rice leaves was 92% higher than manual inspections. When using the Xiaomi Mi5 smartphone, the average running time for processing leaf images under field conditions was 248 milliseconds.

To date, mobile applications for the detection of rice panicles in different growth stages and the recognition of rice growth stages are still poorly explored. This study selected YOLOv8, the newly developed YOLO series (Varghese and Sambanth, 2024), as the base network and investigated lightweight methods. An Android application named YOLO-RPD (YOLOv8-Rice Panicle Detection) was developed to detect rice panicles at three growth stages, including the booting stage, heading stage, and filling stage, and recognize rice growth stages. YOLOv8 and the lightweight YOLOv8 were trained and converted into Neural Network Computing Nibrary (NCNN) models. These models were employed for rice panicle detection on Android smartphones and evaluated using comprehensive evaluation metrics. Our research fills the literature gap related to the usage of deep learning on mobile phones as a platform for rice phenotype applications and provides an easy-to-use application tool for rice growers. Through the easy use of an application for the detection of rice panicles and recognition of rice growth stages, rice growers can facilitate timely precision field management and pursue maximum grain yield.

## 2 Materials and methods

### 2.1 Image acquisition and dataset construction

#### 2.1.1 Image acquisition

The rice images were acquired from two comprehensive field experiments of double cropping rice, namely Exp. 1 and Exp. 2, located in Shapu Research Center, Zhaoging City, Guangdong Province, at 23.16° North latitude and 112.66° East longitude. There were 90 planting plots in Exp. 1 and Exp. 2, respectively. Each plot was 10.8 m × 3.5 m. The experimental plots were designed with different strategies for plant growing practices to ensure diverse rice panicle phenotypes. Different growing strategies, including three cultivars, five levels of N fertilizers (N0-N4), and two planting densities, were applied in each plot, and the experimental plot was designed with three replications. Detailed information of the growing practice in each plot is shown in **Table 1**.

**Table 1:** Different strategies for plant growing practices and the dates of image collection.

	Strategies for plant growing practices			Date of image collection		
	Cultivar	Planting densities/cm2	N fertilizers/kg/ha	Booting stage	Heading stage	Filling stage
Exp. 1	Huahang No.51; Huahang No.57; Guang8you2156	30×14; 30×21;	N0:0; N1:45; N2:90; N3:180; N4:270;	17 June 2021	28 June 2021	11 July 2021
Exp. 2	Y liangyou3089; Huahang No.57; Guang8you2156	30×14; 30×21;	N0:0; N1:90; N2:180; N3:270; N4:360;	11 October 2021	18 October 2021	26 October 2021

**Figure 1:**
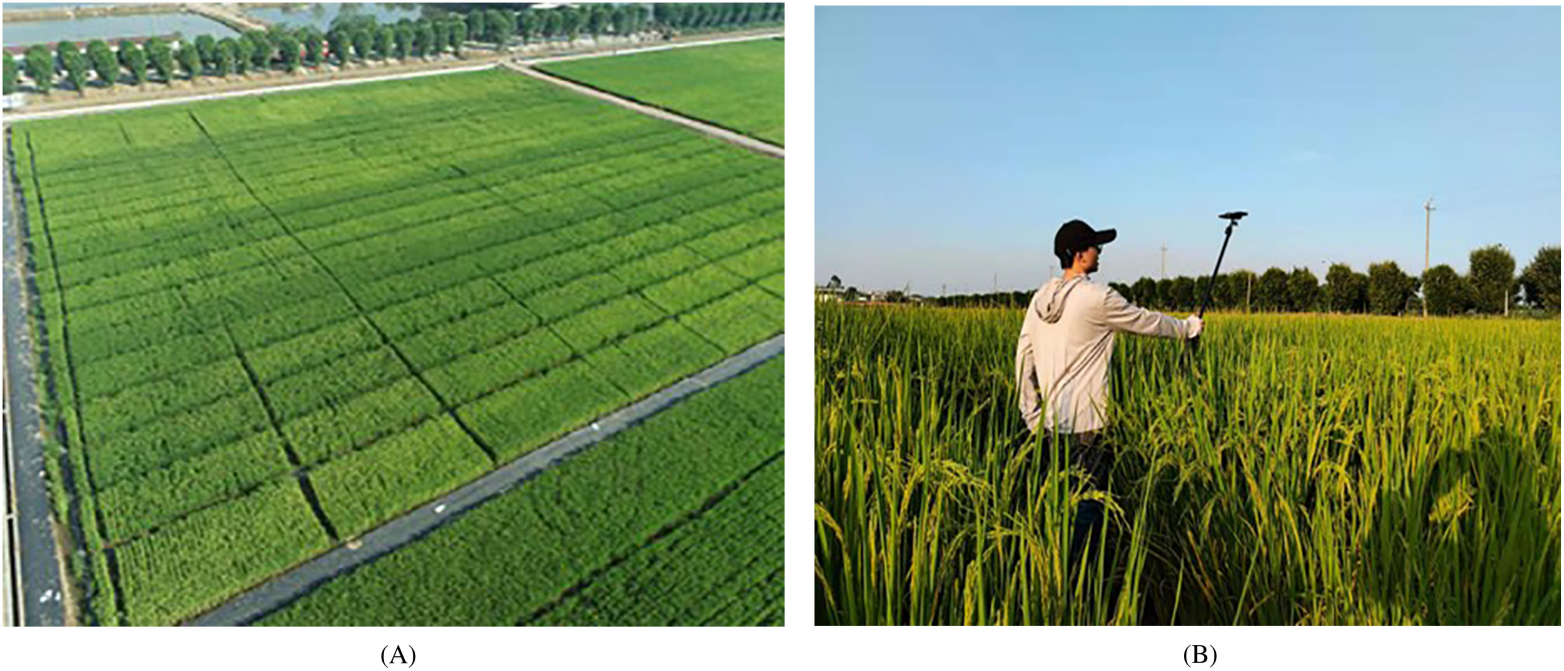
Field experiments. **(A)** Field plot experiments, **(B)** image acquisition.

Rice panicle images were collected using a smartphone, XiaoMi 11, with a 20 million-pixel rear lens, and the images were stored in JPG format with an image resolution of 5792 × 4344 pixels. **Figure 1A** shows the field plot planting experiment, and images were collected from three to five different regions in each planting plot. The researcher held a shooting rod with the smartphone fixed on it. The smartphone was kept at a distance of 1.6 meters above the ground with its rear camera facing down to capture the canopy of rice panicles, as illustrated in **Figure 1B**. To obtain diverse rice panicle phenotypes, first, different shooting angles, including a top view and a side view, were employed in the experiment. In addition, images were collected at booting, heading, and filling stages in both Exp. 1 and Exp. 2. Furthermore, the image acquisition was conducted during natural lighting conditions in the morning (between 8:00 am and 11:00 am) or in the afternoon (between 2:00 pm and 5:00 pm) to enhance the diversity of data. Finally, rice panicle images were captured under different weather conditions, including sunny, cloudy, and rainy days.

#### 2.1.2 Dataset construction

To make the collected rice images suitable for deep learning model training, data preprocessing is an essential step. Typically, data preprocessing consists of three main steps: image cropping, manual annotation, and data augmentation.

First, to improve model training efficiency, the original collected RGB rice images were cropped into five sub-images with small sizes. First, the original image was divided into four sub-images along the horizontal and vertical center lines. Each original image had a size of 5792×4344 pixels, while the cropped sub-images had a size of 2896×2172 pixels. An additional sub-image was cropped around the center point of the images with a size of 2896 × 2172 pixels. Therefore, the number of images in each growth stage after cropping was expanded five times compared to that of the original images.

Second, labelImg, an open-source image annotation software, was used to label the ground truth of the rice panicles with a minimum external rectangle. The manual annotation data are stored in an annotation format.txt file, which contains the coordinate and category information of the annotation box in each image. Each annotation box was recorded with the coordinates of the center point (*x*_min_ *, y*_max_) of the bounding box and the width and height (*w,h)* of the bounding box to determine the relative position of the rice panicle targets in the image. Since rice panicles in different growth stages differ in appearance, different color boxes were used to label the different growth stages of rice panicles. For example, the red box, black box, and blue box were used to label the rice panicle of the booting stage, heading stage, and filling stage, respectively.

Third, to improve the generalization ability of the deep learning model, online data augmentation was used in the experiment with the application of various transformations to the images during the training process, increasing the diversity of the image samples. Five methods were used for data augmentation, including horizontal flipping, vertical flipping, blurring, random changes to hue and saturation, and random changes to brightness and contrast.

Finally, rice panicle images collected at three growth stages in Exp. 2 formed the training and validation sets, while the rice panicle images collected at three growth stages in the Exp. 1 formed the test sets. Specifically, 433, 328, and 323 original images were collected at the booting, heading, and filling stages in Exp.2, respectively, resulting in a total of 1,084 images. The image cropping process resulted in each original image being cropped into five sub-images, with 2,165, 1,640, and 1,615 sub-images obtained for each growth stage, respectively, resulting in a total of 5,420 sub-images. The cropped images were divided into training and validation sets according to an 8:2 ratio, with 4,336 and 1,084 sub-images for training and validation, respectively. Independent test sets were collected from three growth stages in Exp. 1, with 40 original images collected at each growth stage, resulting in a total of 120 original images. After image cropping, the test set contained a total of 600 sub-images.

### 2.2 Construction of rice panicle detection model based on lightweight YOLOv8

YOLOv8 is a regression-based one-stage target detection algorithm that integrates various optimization strategies to balance speed and accuracy. YOLOv8 is divided into s, x, l, m, and n versions. They have similar network structures with different depths and widths. YOLOv8n has the smallest structure and the shallowest depth and thus has the fastest running speed. In this study, YOLOv8n was selected as the base network to achieve fast and accurate detection of rice panicles in a complex field environment. This study further investigated the improvement method of introducing a lightweight network to make it more suitable for deployment on mobile devices with low computing power.

#### 2.2.1 YOLOv8n

**Figure 2:**
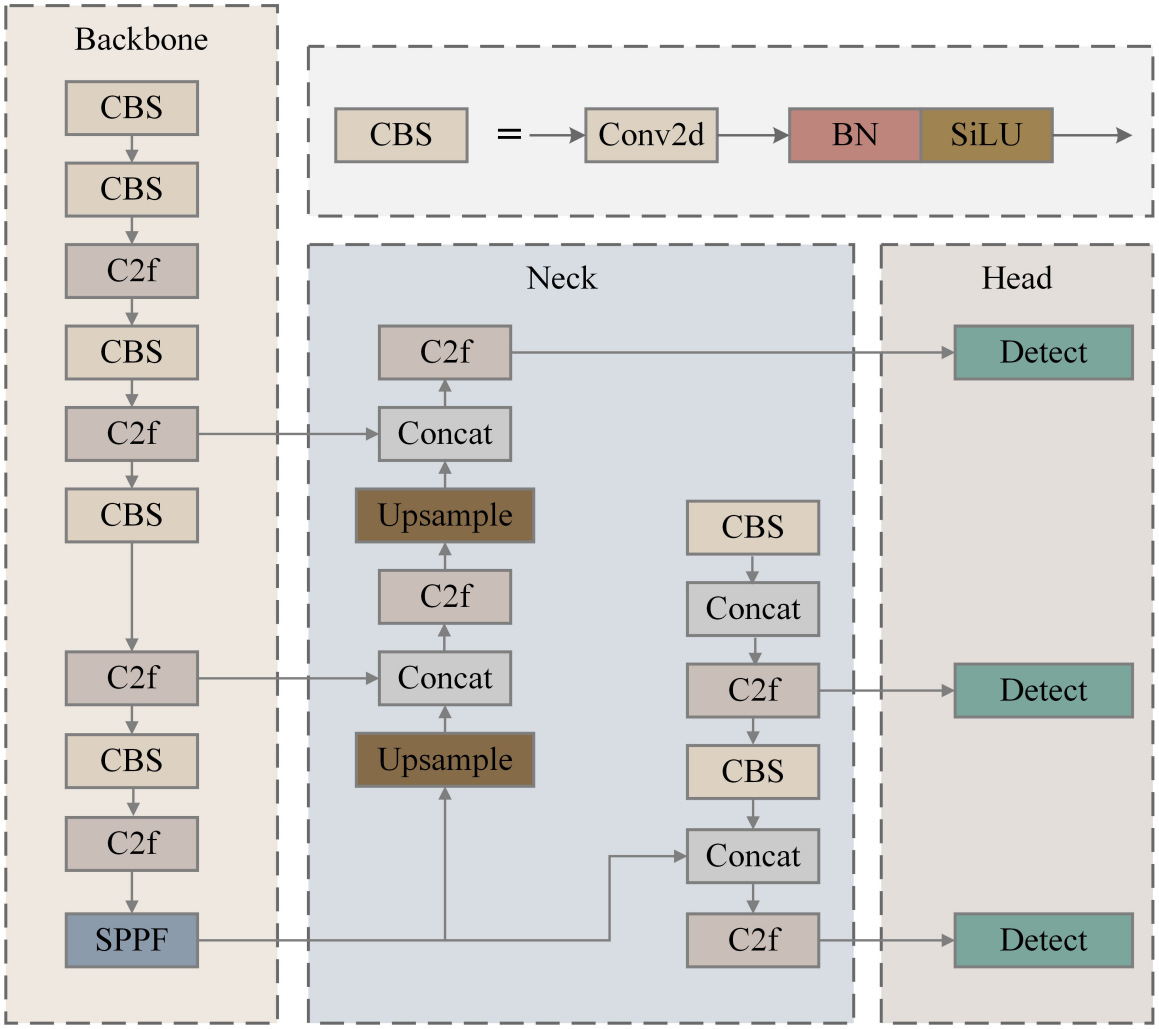
Structure of YOLOv8n (You only look once).

The YOLOv8n network structure consists of four parts: input, backbone, neck, and prediction, as shown in **Figure 2**. The input uses mosaic data enhancement, Mixup, Random Perspective, and HSV (Hue, Saturation, Value) Augment to process the dataset. The CSPDarknet53 was adopted as the backbone for feature extraction and aggregation. The neck includes a feature pyramid network (FPN) and a pyramid attention network (PAN) to fuse the shallow features extracted from the backbone with the original depth features to improve feature extraction and pass the image features to the final detection prediction layer. The prediction achieves target detection and category prediction using the GIOU Loss function.

The backbone component primarily focuses on feature extraction from images and utilizes the CSPDarknet-53 network architecture, which comprises CBS, C2f, and SPPF (Spatial Pyramid Pooling-Fast) modules.

The backbone of the CSPDarknet53 network in YOLOv8 consists of several key components, including the CBL, C2f, and SPPF modules, as illustrated in **Figure 2**. The CBL module is fundamental to the YOLOv8 architecture, integrating convolutional layers, batch normalization, and the SiLU activation function to facilitate effective feature extraction and dimensionality reduction. It incorporates more skip connections and additional split operations, enabling superior feature information transmission. This module adopts design principles from both Bottleneck and ELAN, allowing for effective feature transformation and fusion while addressing convergence issues associated with deeper networks. The SPPF module is strategically positioned between the feature extraction and feature fusion layers. It begins by halving the number of input channels through standard convolution and subsequently divides the output into four branches. Max pooling is applied to generate feature maps at different scales, followed by a concatenation operation that doubles the input channel count. By utilizing three 5 × 5 convolution kernels, SPPF significantly reduces computational load while enhancing detection efficiency, achieving speeds that are approximately twice as fast as the previous SPP module used in previous-generation algorithms.

#### 2.2.2 YOLO_ECO construction

##### 2.2.2.1 The improvement of backbone

**Figure 3:**
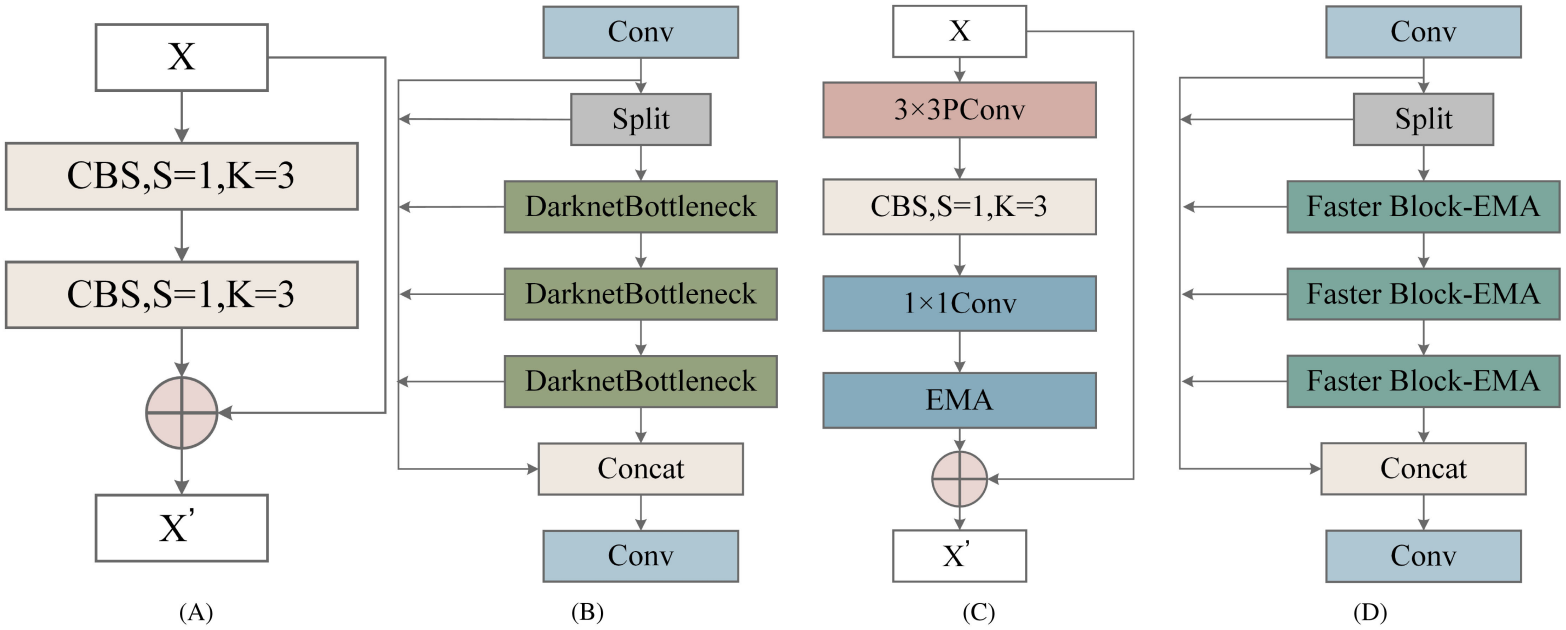
Structure of Cross-Stage Partial Network (C2f) and C2F-Faster- Effective Multi-scale Attention (C2F-Faster-EMA) Modules: **(A)** Bottleneck structure, **(B)** C2f module, **(C)** Faster Block- Effective Multiscale Attention (Faster Block-EMA) structure, **(D)** C2F-Faster-EMA module.

In this study, the lightweight module C2f-Faster Block-Effective Multi-scale Attention (C2f-Faster-EMA) was used to replace C2f of the original YOLOv8n backbone network. The structures of C2f and C2F-Faster-EMA are illustrated in **Figure 3**. The C2F-Faster-EMA structure was inspired by the Partial Convolution (PConv) concept from FasterNet. This modification aimed to reduce computational load. In the C2F-Faster-EMA module, convolution was applied to only 1*/*4 of the input channels, while the remaining 3*/*4 remain unchanged. The convolved channels were then concatenated with the unprocessed channels, ensuring that the output retains the original number of channels and feature map dimensions. This design minimized redundant computations while preserving essential channel information. Although 3*/*4 of the channels were not convolved, subsequent CBS operations and 1 × 1 convolutions effectively extract useful information, enhanced by the Effective Multi-scale Attention (EMA) module (Ouyang et al., 2023).

**Figure 4:**
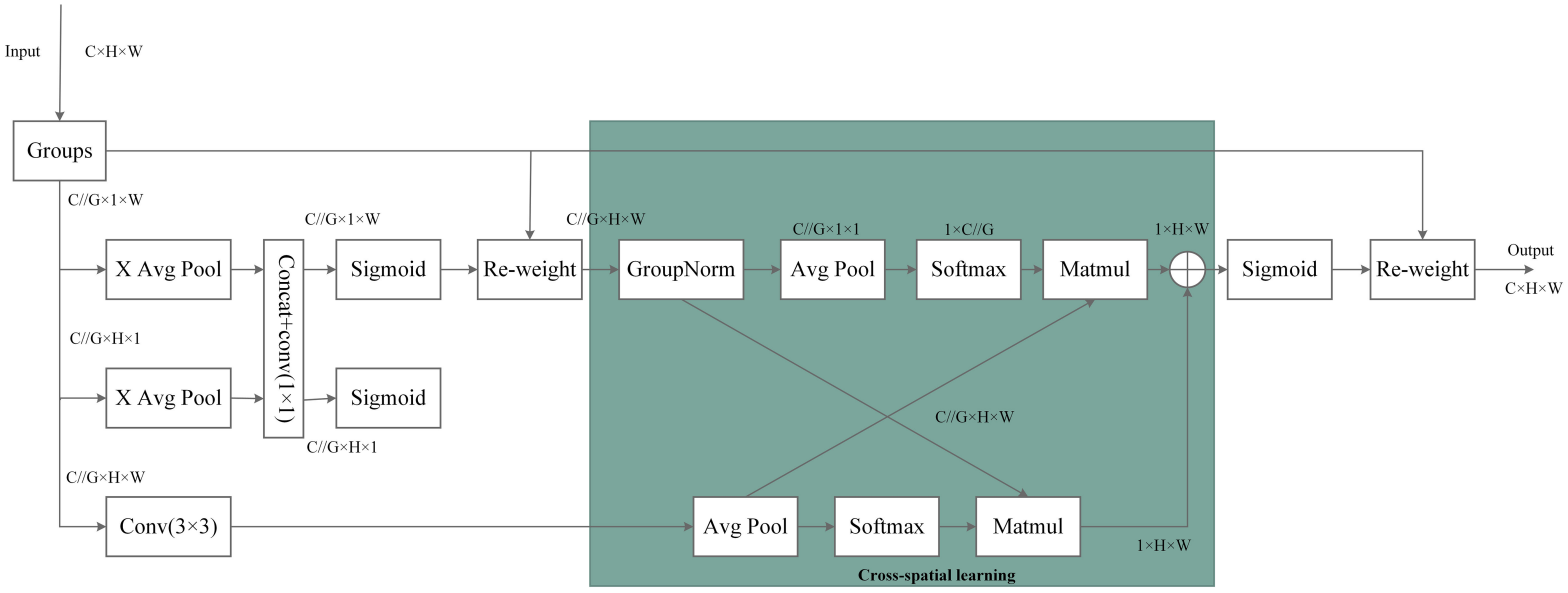
Structure of Effective Multi-scale Attention (EMA).

The structure of EMA is illustrated in **Figure 4**. The EMA module is designed to preserve channel-specific information while reducing computational overhead. It reshapes some channels into the batch dimension and divides the channel dimension into multiple sub-features, ensuring that spatial semantic features are evenly distributed within each feature group. Additionally, by encoding global information to recalibrate channel weights in each parallel branch, the outputs from the two parallel branches are further aggregated through cross-dimensional interactions to capture pixel-level paired relationships. The EMA module employs two parallel sub-networks: a 1 × 1 branch and a 3 × 3 branch. The 1 × 1 branch is used to extract global information from channel features, while the 3 × 3 branch captures dependencies between local features. By performing cross-space learning on the outputs of these two branches, EMA effectively aggregated global and local features and models long-range dependencies across different scales.

In YOLOv8’s C2f module, the Bottleneck was replaced by C2F-Faster-EMA. The first CBS module outputs 2 × c channels, which are split into two parts of c channels each and stored in a list. Each C2FFaster-EMA module processes the last element of this list, storing its output back into y for the next module. After passing through n C2F-Faster-EMA modules, the elements in the list were concatenated along the channel dimension, resulting in a (2 + n) × c feature map, which was then compressed to c 2 channels via the second CBS module.

While YOLOv8 initially utilized multiple Bottleneck modules to fuse multi-scale features, which improved feature expressiveness and detection accuracy, each addition increased computational overhead. By replacing Bottlenecks with C2F-Faster-EMA, we significantly reduced computational complexity, as each Faster Block only convolves 1*/*4 of the channels, thereby lowering the cost of subsequent 1 × 1 convolutions. This approach, validated by experimental results, demonstrated a substantial improvement in inference speed in YOLOv8.

##### 2.2.2.2 The improvement of the neck structure

The neck structure of YOLOv8 employs standard convolution and the C2f module, effectively integrating low-level detailed features with high-level abstract features. However, this mechanism increases the model’s parameter count and computational burden. Therefore, this paper introduces an innovative network architecture, SlimNeck, to alleviate the complexity of the model while maintaining accuracy (Li et al., 2024).

**Figure 5:**
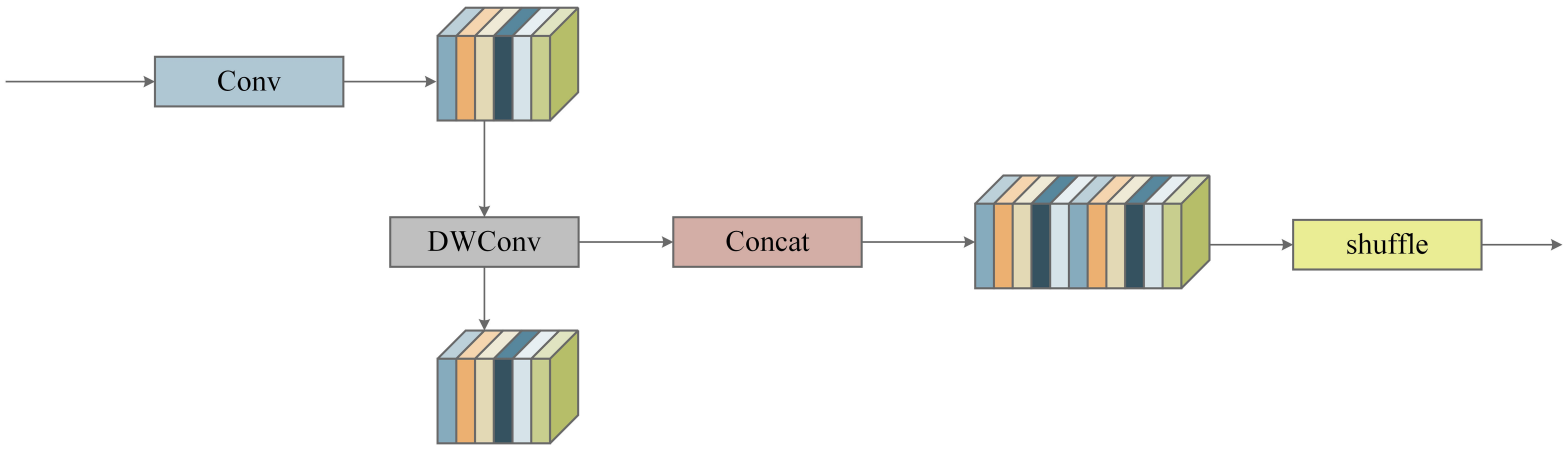
Structure of Group Shuffle Convolution (GSConv).

SlimNeck comprised the GSConv module and the cross-stage partial network module VoV-GSCSP. The design of the GSConv module first reduces the channel count by half through standard convolution, followed by further processing with Depthwise Separable Convolution (DSConv), and subsequently combines the outputs using a Concat module. The structure of GSConv is shown in **Figure 5**. Finally, a shuffle module is applied to distribute local feature data evenly among different channels, thereby restoring the initial channel count. This approach minimizes computational overhead while preserving rich channel information, with the computational cost of GSConv being approximately 60%−70% that of standard convolution, resulting in a savings of 30%−40% in computational resources.

**Figure 6:**
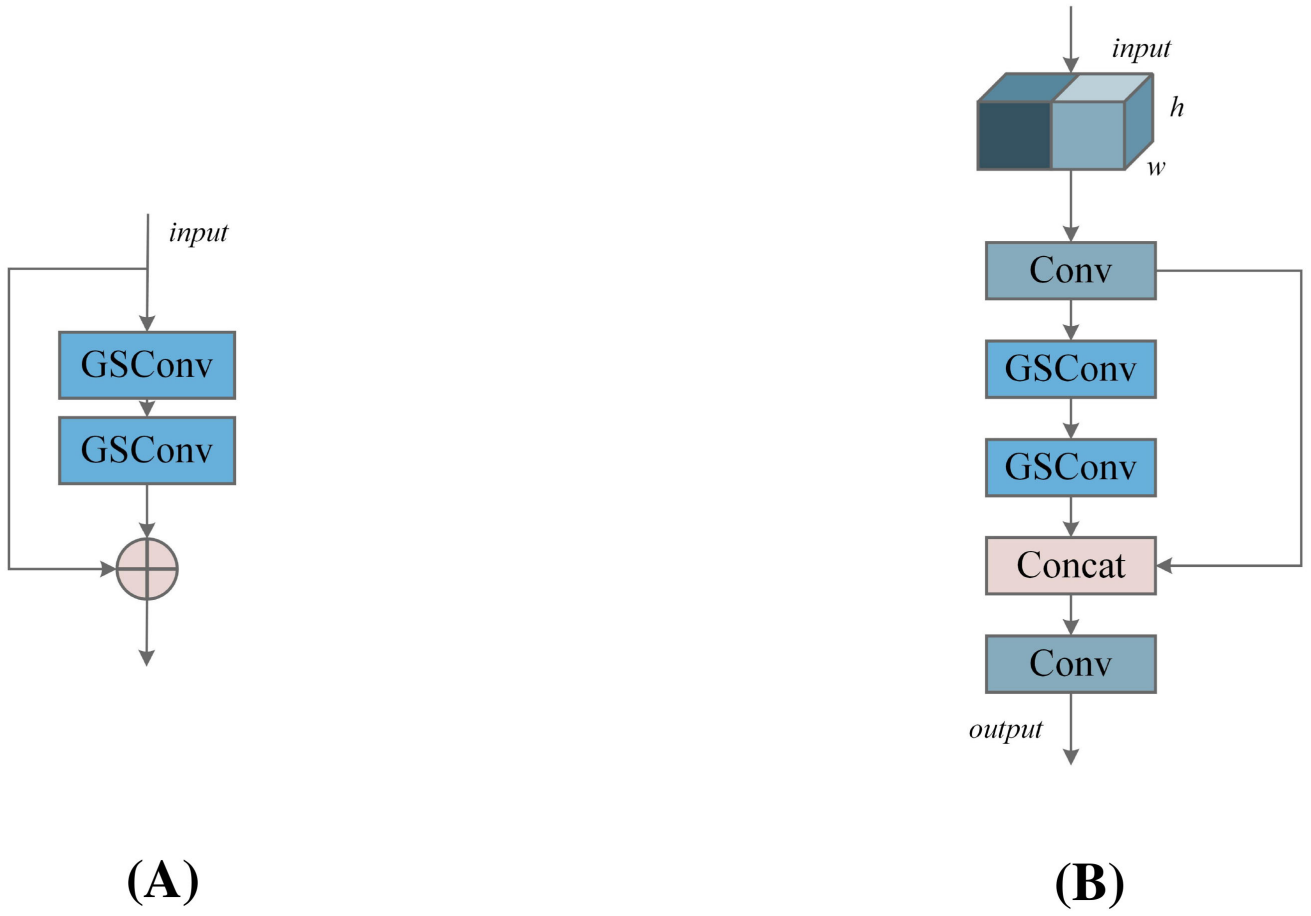
**(A)** Group Shuffle (GS) Bottleneck module, **(B)** VoV-Group Shuffle Cross Stage Partial (VoVGSCSP) module.

The VoV-GSCSP module further reduces computational and architectural complexity while preserving model accuracy. By replacing traditional CSP (standard convolution) with VoV-GSCSP, the average floating-point operations (FLOPs) are reduced by 15.72%, significantly enhancing computational efficiency. As shown in **Figure 6A**, this structure divides the input feature information into two streams: one stream is processed through 1×1 convolution, while the other stream undergoes processing via the GS Bottleneck. The outputs from these two streams are then concatenated and subjected to further convolution to modify the output channels. This design enhances the mixing and cross-flow of gradients from different locations, thereby improving the learning capability of the network.

By adopting the lightweight convolution GSConv to replace standard convolution, we used a one-shot aggregation method to design the VoV-GSCSP module, as shown in **Figure 6B**. The Concat module is employed to connect features from different stages, facilitating the fusion of features across layers. Compared to standard convolution, GSConv captures richer feature representations with fewer parameters. By expanding the receptive field and increasing network depth, the VoV-GSCSP structure generates deeper feature maps, enhancing feature extraction capability.

In summary, the design of SlimNeck not only enables efficient feature extraction and fusion but also ensures a significant reduction in computational complexity while maintaining high detection accuracy. This allows the YOLO-RPD model to achieve commendable lightweight performance while preserving excellent detection precision.

##### 2.2.2.3 The improvement of the detection head

**Figure 7:**
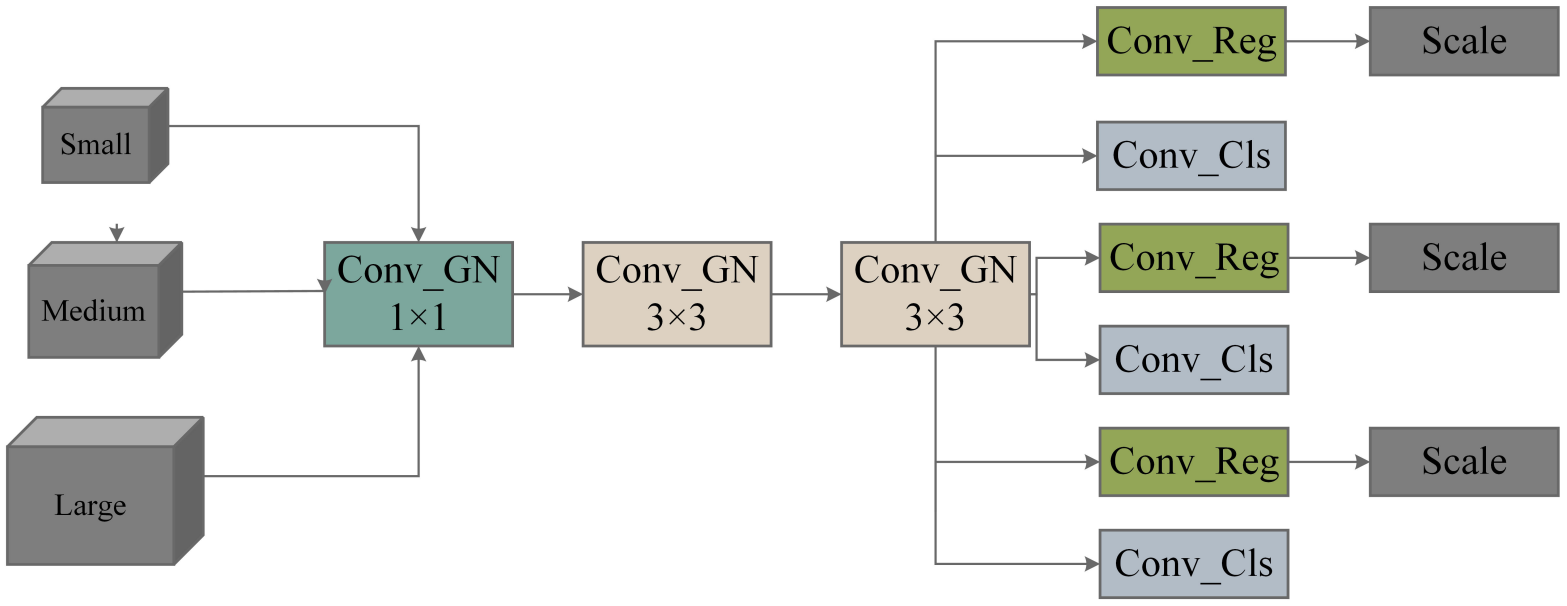
Illustrates the structure of LSCD (Lightweight and Scalable Convolutional Detector).

We present a lightweight detection head called the Lightweight Shared Convolutional Detection (LSCD), with its network architecture illustrated in **Figure 7**. A, effectively leverages the advantages of Group Normalization (GroupNorm) and shared convolution to minimize computational load and complexity while maintaining efficient feature integration.

GroupNorm divides the input image, sized N, C, H, and W, into several groups, computes the variance and mean for each group, and normalizes all data within that group. Since GroupNorm’s calculations depend on the number of channels C rather than the batch size N, it is particularly useful in scenarios where memory is constrained or when the sample size is small.

**Figure 8:**
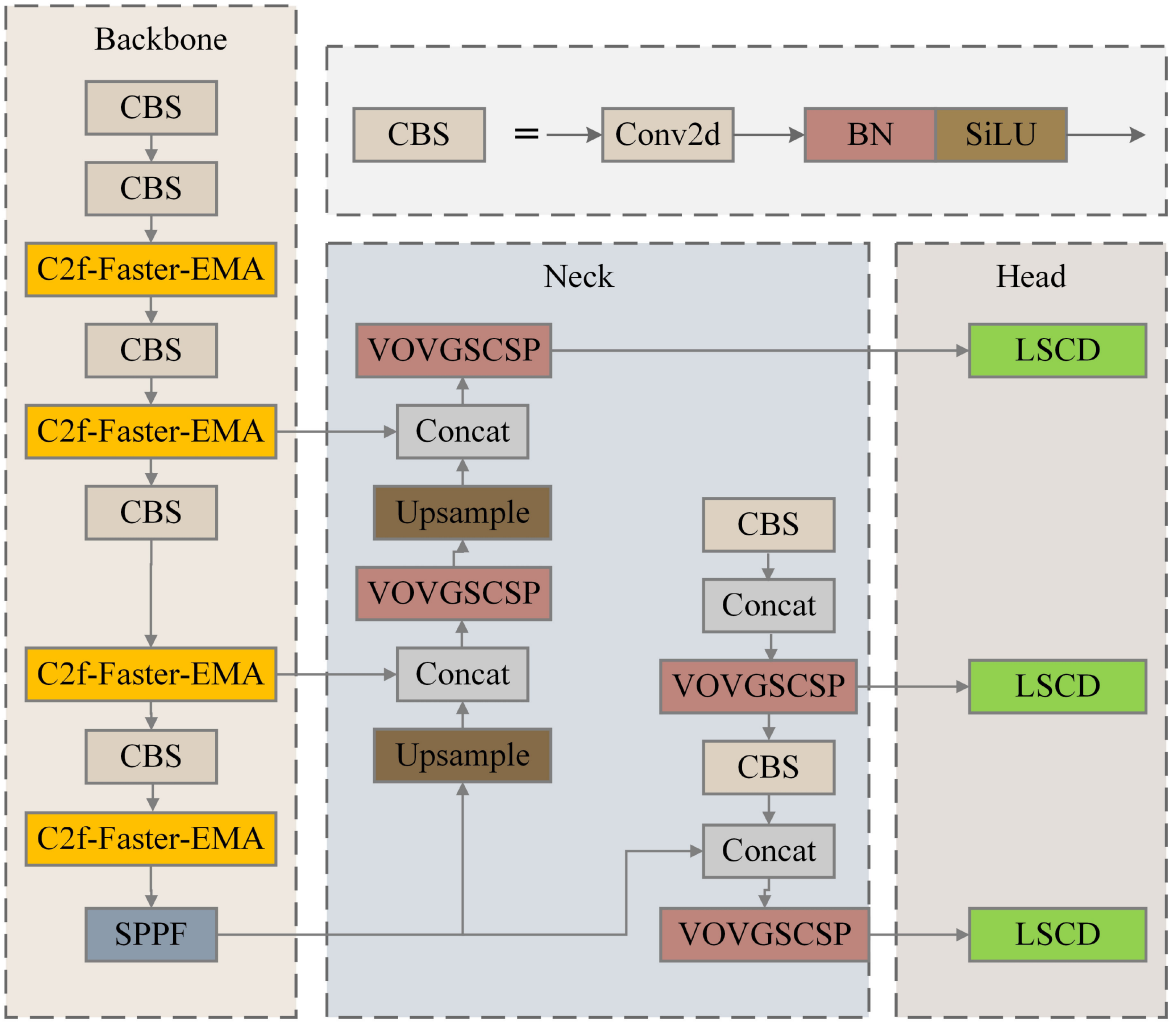
Structure of YOLO-Efficient Computation Optimization (YOLO_ECO), the lightweight YOLOv8.

Shared convolution is a core concept in CNNs that allows the same convolution kernel to apply identical weight parameters across different spatial locations. This mechanism significantly reduces the number of parameters that need to be trained, enhances computational efficiency, and lowers the risk of overfitting. Shared convolution effectively extracts local features while preserving spatial structural information, thereby improving the model’s generalization ability. By efficiently extracting local features while preserving spatial structure, shared convolution enhances the model’s generalization ability. The Conv GN 1 × 1 module represents the combination of Group Normalization and convolution, with a kernel size of 1×1. Two yellow Group Normalization convolution modules (Conv GN 3×3) share weights, while three blue prediction box convolution modules (Conv Box) and three red classification convolution modules (Conv Cls) also share weights. Each Conv Box module is followed by a Scale module, which is used to match the detection of targets at different scales. The structure diagram of the improved YOLO_ECO is shown in **Figure 8**.

### 2.3 Design of the Android platform

#### 2.3.1 NCNN

NCNN, an open-source high-performance neural network forward computing framework developed by Tencent Youtu Lab, is optimized for mobile devices. This framework has achieved exceptional computational speeds on mobile CPUs, supports multiple platforms, including Android and iOS, and is devoid of third-party dependencies, facilitating integration. NCNN supports a variety of mainstream convolutional neural network architectures, including VGG, ResNet, and YOLOv8, and is characterized by low memory usage and a compact library size, with the entire library being less than 500 K in size. It also supports 8-bit quantization and custom layer extensions, offering a user-friendly model conversion tool that simplifies the application of deep learning models in practical scenarios. Due to its efficiency and flexibility, NCNN has been extensively applied in the domains of image classification and object detection. First, the parameters of the YOLOv8 algorithm structure are adjusted to meet the requirements of mobile software development. Subsequently, the export.py tool provided by the YOLO algorithm is utilized to generate a torchscript format file, facilitating the transfer and storage of the trained model across different frameworks. Following this, parameter files (.param) and compiled binary files (.bin) are generated through PyTorch Neural Network exchange (PNNX), with certain parameters undergoing correction. Finally, the corrected parameters are integrated into the NCNN framework within Android Studio, connecting to a Xiaomi smartphone running a customized MIUI system based on the Android operating system.

#### 2.3.2 YOLO-RPD software development

**Figure 9:**
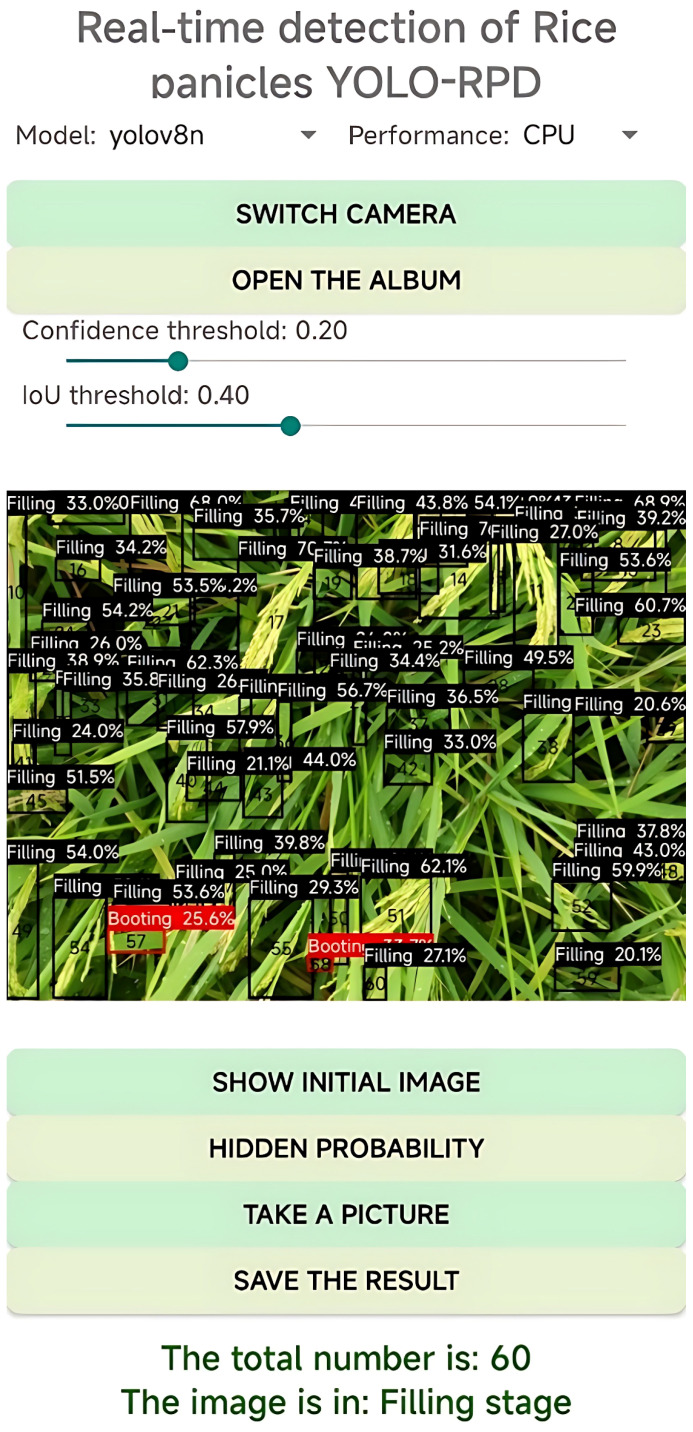
Interface of the YOLO-Rice Panicle Detection (YOLO-RPD).

This paper developed an Android application called YOLO-RPD for real-time detection of rice panicles and recognition of rice growth stages. The application integrates a lightweight deep learning model, the YOLO_ECO, and different models for rice panicle detection, which can efficiently run offline or online on Android devices. The software design of YOLO-RPD consists of four functional modules: image selection, rice panicle detection, result display, and real-time detection. The application interface is shown in **Figure 9**.

In the image selection module, rice panicle images were first selected on the mobile phone through the photo album and were displayed in the middle of the main interface. To adapt to different usage scenarios, different detection deep learning models can be quickly switched using the radio buttons. First, YOLOv8 and lightweight YOLOv8 (YOLO_ECO) can be chosen, and then input image sizes can be adjusted to the fixed resolution size of 1280 × 1280 pixels or 640 × 640 pixels. Moreover, different quantization models, namely FP32, FP16, and INT8, can be selected. The results display module displays predicted bounding boxes of rice panicles in the image, and the output log of the results is also shown. The output log includes the number of detected rice panicles, the number of detected rice panicles in different growth stages, the rice growth stage, and the running time. In particular, the color of the predicted bounding boxes represents the growth stages of the rice panicles. In our study, the predicted boxes with the most colors indicate the growth stages in the rice images. The user can click on the save result button to save the predicted image to their phone’s photo album and zoom in to check the model’s performance. The users can also click on the hidden probability button to hide the growth stage label and click show initial image to see the original picture. In the real-time detection module, users can use the camera of the Android phone to acquire the rice panicle images and perform real-time detection of the rice panicles in the field. The predicted bounding boxes of rice panicles in the image and the output log of the results are displayed on the screen. The confidence threshold and IoU threshold can be modified to observe the different effects on the performance of rice panicle detection.

### 2.4 Experimental platform

In this study, YOLOv8n, one of the YOLOv8 series, and the lightweight YOLOv8n (YOLO_ECO) were used as the base models, and a transfer learning approach was employed to train a lightweight model suitable for real-time detection of rice panicles. In this experiment, a computer with a Intel i7-13700K CPU @ 4.85 GHz processor, NVIDIA GeForce RTX 4090Ti GPU (11GB video memory), 64 GB memory, and 2 TB mechanical hard disk was used. The operating system was Windows 10 64-bit. The programming framework was Python 3.8, and the deep learning framework was PyTorch1.8.1.

In order to evaluate the influence of different image sizes on model acceleration, two different image sizes (1280 pixels and 640 pixels) were used in this study. During training, the optimizer uses Stochastic Gradient Descent (SGD) and cosine learning rate attenuation strategies. The network training parameter settings include an initial learning rate of 0.01, a momentum of 0.937, a weight attenuation of 0.00005, a batch size of 16, a step size of 150, and a learning rate of 0.1.

### 2.5 Evaluation metrics

In deep learning, evaluation metrics are the standards used to evaluate the performance of models. Among them, precision and recall are two commonly used evaluation metrics, and their calculation formulas are shown in Equations 1 and 2, respectively. The average precision (*AP*) and mean average precision (mAP) are also used to assess the performance of the models. The *AP* value represents the area under the precision-recall (*P* − *R*) curve, ranging from 0 to 1. The higher the *AP* value, the better the accuracy and performance of the model, and the calculation formula is shown in Equation 3. The mAP is the average of the AP values for the three growing stages and is used as the final performance indicator, with a calculation formula shown in Equation 4. FLOPs is an important metric for evaluating the performance of processors or neural network models. It represents the total number of floating point operations required for a single forward pass of the model. Lower FLOPs typically mean faster inference, making the model more suitable for deployment on resource-constrained devices such as mobile or embedded systems.


(1) $$P_{S}=\text{Precision }(S)=\frac{TP}{TP+FP}$$

(2) $$R_{S}=\text{Recall }(S)=\frac{TP}{TP+FN}$$

(3) $$AP_{S}=\int_{0}^{1}P(R_{S})\;dR_{S}$$

(4) $$mAP=\frac{1}{3}\sum_{1}^{3}AP_{S}$$

where *S* represents the three growth stages of rice: booting stage, heading stage, or filling stage. *TP* stands for true positive; *FP* stands for false positive, and *FN* stands for false negative.

## 3 Results and discussion

### 3.1 Data augmentation for enhanced model performance

To enhance our model’s detection performance in complex environments and improve its generalization capability, we introduced various data augmentation techniques. These techniques—mosaic, brightness increase, grayscale conversion, and flipping—were designed to simulate diverse real-world scenarios and bolster the model’s robustness. We set up five experimental groups, each incrementally incorporating different augmentation methods. The results are shown in **Table 2**. The optimal detection performance was achieved in Group E, where the mAP reached 87.2%. Specifically, as augmentation techniques accumulated, detection accuracies for the booting, heading, and filling stages of rice panicles increased from 89.2%, 85.2%, and 69.3% in Group A to 94.0%, 90.3%, and 77.3% in Group E, respectively. The results demonstrate that a well-considered combination of augmentation strategies can significantly enhance the model’s performance under challenging conditions such as low light and occlusion. This approach provides an effective means to improve the model’s generalization ability.

**Table 2:** Test results of YOLO_ECO trained on different datasets.

Experimental group	Combination of training sets	AP(%)		
		Booting	Heading	Filling
A	Original images + mosaic	89.2	85.2	69.3
B	A + Increase brightness	90.3	86.8	71.7
C	B + Grayscale	91.7	87.6	75.8
D	C + Flipping	92.5	88.2	76.2
E	All augmentations combined	94.0	90.3	77.3

### 3.2 Ablation studies

To assess the efficacy of the proposed improvements in this study, four sets of ablation experiments were conducted in the rice panicle dataset. The original YOLOv8n network served as the baseline, with experimental conditions and training parameters kept consistent throughout the evaluation. The validation criteria encompass mAP, the number of parameters, FLOPs, and the model size. The experimental results are presented in **Table 3**, where a ‘√’ denotes the implementation of the corresponding method. To control the variables, the number of epochs for all models was set to 150.

**Table 3:** Performance evaluation of four deep learning models for rice panicle detection.

Model	C2f-Faster EMA	Slim Neck	LSCD	Network Layers	Parameters (M)	mAP@0.5 (%)	FLOPs (G)	Model size(M)
YOLOv8n	–	–	–	168	3	85.2%	8.1	6.2
Strategy 1	✓			317	1.6	85.4%	6.3	3.6
Strategy 2		✓		244	2.8	86.1%	7.4	5.8
Strategy 3			✓	202	2.4	85.6%	6.6	5.4
Strategy 4	✓	✓		300	2.4	86.7%	6.2	3.5
Strategy 5	✓	✓	✓	484	1.8	87.2%	4.1	3.1

YOLOv8n, as the baseline model, has 168 network layers, 3 M parameters, a mAP@ 0.5 of 85.2%, FLOPs of 8.1 G, and a model size of 6.2 MB. Strategy 1 introduces the C2f-Faster-EMA module, which increases the mAP@0.5 to 85.4%, a 0.2 percentage point improvement over YOLOv8n. Furthermore, FLOPs decreases to 6.3 G, the parameter count is reduced to 1.6 M, and the model size is significantly reduced to 3.6 MB, a 41.9% reduction compared to YOLOv8n. Strategy 2 introduces the SlimNeck module, achieving an mAP of 86.1%, a 0.9 percentage point improvement over YOLOv8n. FLOPs decrease to 7.4 G, while the parameter count is 2.8 M, marking a 6.5% reduction compared to YOLOv8n. Strategy 3 integrates the LSCD module, reaching an mAP of 85.6%, a 0.4 percentage point improvement over the baseline. This strategy maintains efficiency with FLOPs at 6.6 G and a model size of 5.4 MB, which is a 12.9% reduction compared to YOLOv8n, alongside having 2.4 M parameters. Strategy 4 combines C2f-FasterEMA and SlimNeck, with the model having 2.4 M parameters and an mAP of 85.6%, a 0.4 percentage point improvement over YOLOv8n. FLOPs is 6.2 G, and the model size is further reduced to 3.5 MB, a 43.8% reduction compared to YOLOv8n. Strategy 5 integrates C2f-Faster-EMA, SlimNeck, and LSCD, reducing the parameter count to 1.8 M, FLOPs to 1.8 G, and achieving an mAP of 87.2%, which is a 2 percentage point improvement over YOLOv8n. FLOPs is reduced by 77.8%, and the model size is further reduced to 3.1 MB, a 50% reduction compared to YOLOv8n. In summary, Strategy 3, the proposed model YOLO_ECO, performs best across all dimensions. Despite an increase in the number of layers, the mAP improves by 2 percentage points to 87.2%, the highest among all strategies. At the same time, FLOPs are significantly reduced by 77.8%, and the model size is reduced by 50%, ensuring efficient inference speed and smaller storage requirements.

### 3.3 Evaluation of different rice panicle detection models

**Figure 10:**
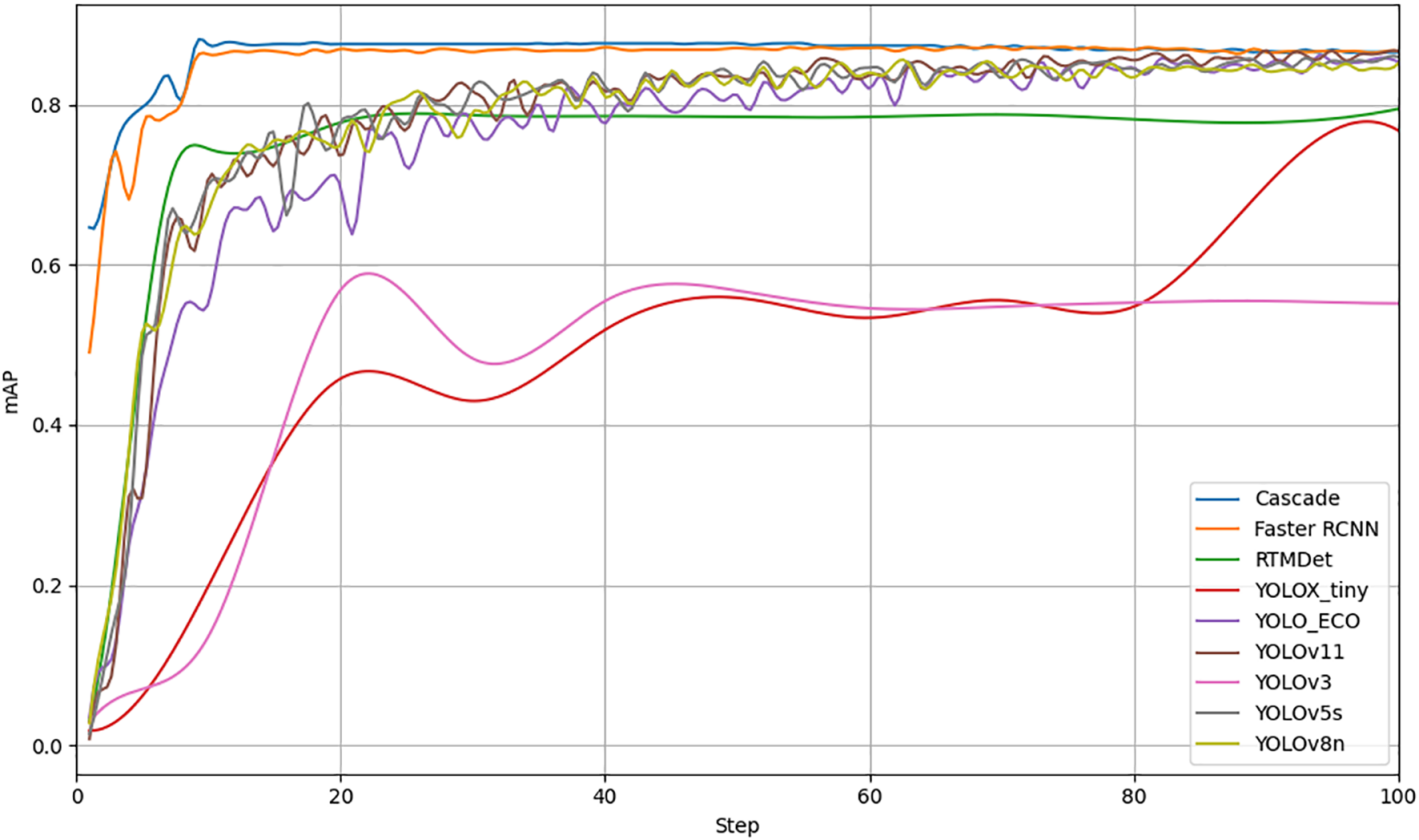
Performance evaluation of eight deep learning models for rice panicle detection.

Deep learning-based object detection models are typically divided into two categories: one-stage and two-stage networks. Two-stage networks, such as Faster RCNN (Faster Region-based Convolutional Neural Network) (Ren et al., 2017) and Cascade (Cai and Vasconcelos, 2018), first generate candidate boxes through an RPN and then predict the location and class of objects. One-stage networks, as RTMDet (Real-Time Multi-Scale Detection) (Zhang et al., 2023) and the YOLO series (Redmon et al., 2016), perform object detection directly. To compare the effectiveness of different deep learning models for detecting rice panicles in field environments, this study selected Cascade, Faster RCNN, RTMDet, and YOLO series models for the performance evaluation. All the models were trained and evaluated over 150 epochs, and the mAP@50 curve is shown in **Figure 10**.

**Table 4** presents the performance of these eight models. The results indicate that the Cascade network achieved the highest mAP value of 86.6% among the two-stage networks; however, it has a parameter count of 69.18 M, FLOPs of 205G, and a model size of 56.1 MB, making it quite large. Among the one-stage networks, YOLOv8n achieved a high mAP of 85.2% with a parameter count of 3.05 M, FLOPs of 8.1 G, and a model size of 6.2 MB, and thus, it remains relatively resource-intensive.

**Table 4:** Performance evaluation of four deep learning models for rice panicle detection.

Model	*mAP*(*/*%)	Parameters (M)	FLOPs (G)	Model size (*/*MB)
Faster RCNN	85.1%	413	178	108
RTMDet	84.1%	48.76	8.033	40.2
Cascade	86.6%	69.18	205	56.1
YOLOv3s	57.4%	61.54	77.46	62.2
YOLOv5s	83.4%	91.12	23.8	18.1
YOLOX_tiny	78.9%	5.03	7.552	71.3
YOLOv8n	85.2%	3.05	8.1	6.2
YOLOv11n	87.3%	2.58	6.3	5.3
YOLO_ECO	87.2%	1.81	4.1	3.1

In contrast, the model proposed in this study demonstrates outstanding performance among all the networks. The proposed model, YOLO_ECO, achieves the highest mAP of 87.2%, 2% and 0.6% higher than YOLOv8n and Cascade, respectively. In addition, YOLO_ECO demonstrated significantly optimized parameter count, computational demand, and model size. Compared to YOLOv5s, our model’s size is reduced by 82.9% (from 18.1 MB to 3.1 MB), parameter count is reduced by 98.0% (from 91.12 M to 1.81 M), and FLOPs is reduced by 82.8% (from 23.8 G to 4.1G). While YOLOv11 improves mAP by 1% over YOLO_ECO, our model significantly enhances efficiency. It reduces parameters by 0.77M (30%), FLOPs by 2.2G (35%), and model size by 40%, highlighting its optimized performance without compromising accuracy. Thus, the proposed model maintains high detection accuracy while substantially lowering computational resource requirements, making it particularly suitable for practical applications in field environments.

### 3.4 Evaluation of rice panicle detection using YOLO-RPD

In this study, an Android smartphone application called YOLO-RPD was developed for rice panicle detection and rice growth stage detection. YOLOv8n and YOLO_ECO were trained and converted to NCNN models, which were then further deployed using different strategies, including input image size and quantization method. The model size and detection performance of different models deployed in YOLO-RPD were comprehensively analyzed. Simultaneously, to distinguish between different deep learning models easily, the models were named after the feature network, quantization method, and rescale size. For example, YOLOv8n_INT8_640 represented a model that used the YOLOv8n network, INT8 quantization method, and a rescale image size of 640.

#### 3.4.1 Comparison of YOLO-ECO and YOLOv8n

As shown in **Table 5**, the most important factor that affects the average precision (AP) was the feature extraction network. YOLO_ECO performed better than YOLOv8n, especially in detecting rice panicles in the filling stage, where images had more complicated rice phenotypes with rice overlapping and adhered scenes. With the same deployment strategy, including an input image size of 1280 and FP32 quantitative model, the proposed model achieved an average precision of 96.4%, 93.2%, and 81.5% in detecting rice panicles in the three growth stages, respectively, which was 2.7%, 3.9%, and 3.4% higher than the YOLOv8n. This resulted in an overall mAP increase of 3.3%.

**Table 5:** Performance evaluation of YOLO-RPD.

Models	Quantitative model	Input image size	AP (/%)			mAP (/%)	Model size (/)MB
			Booting stage	Heading stage	Filling stage		
YOLOv8n	FP32	1280	93.7	89.3	78.1	88.1	12.2
	FP16	1280	92.6	88.0	77.8	86.1	6.1
	INT8	1280	91.4	86.9	77.4	85.2	3.0
	FP32	640	91.9	88.7	74.7	85.1	11.8
	FP16	640	91.3	87.5	73.1	83.9	5.9
	INT8	640	90.5	86.8	72.4	83.2	2.8
YOLO_ECO	FP32	1280	96.4	93.2	81.5	90.4	7.5
	FP16	1280	94.7	91.3	80.9	88.9	4.1
	INT8	1280	93.2	90.4	78.3	87.3	1.9
	FP32	640	94.0	90.3	76.4	86.9	7.1
	FP16	640	93.5	89.4	74.2	85.7	3.6
	INT8	640	92.3	87.2	73.1	83.9	1.7

**Figure 11:**
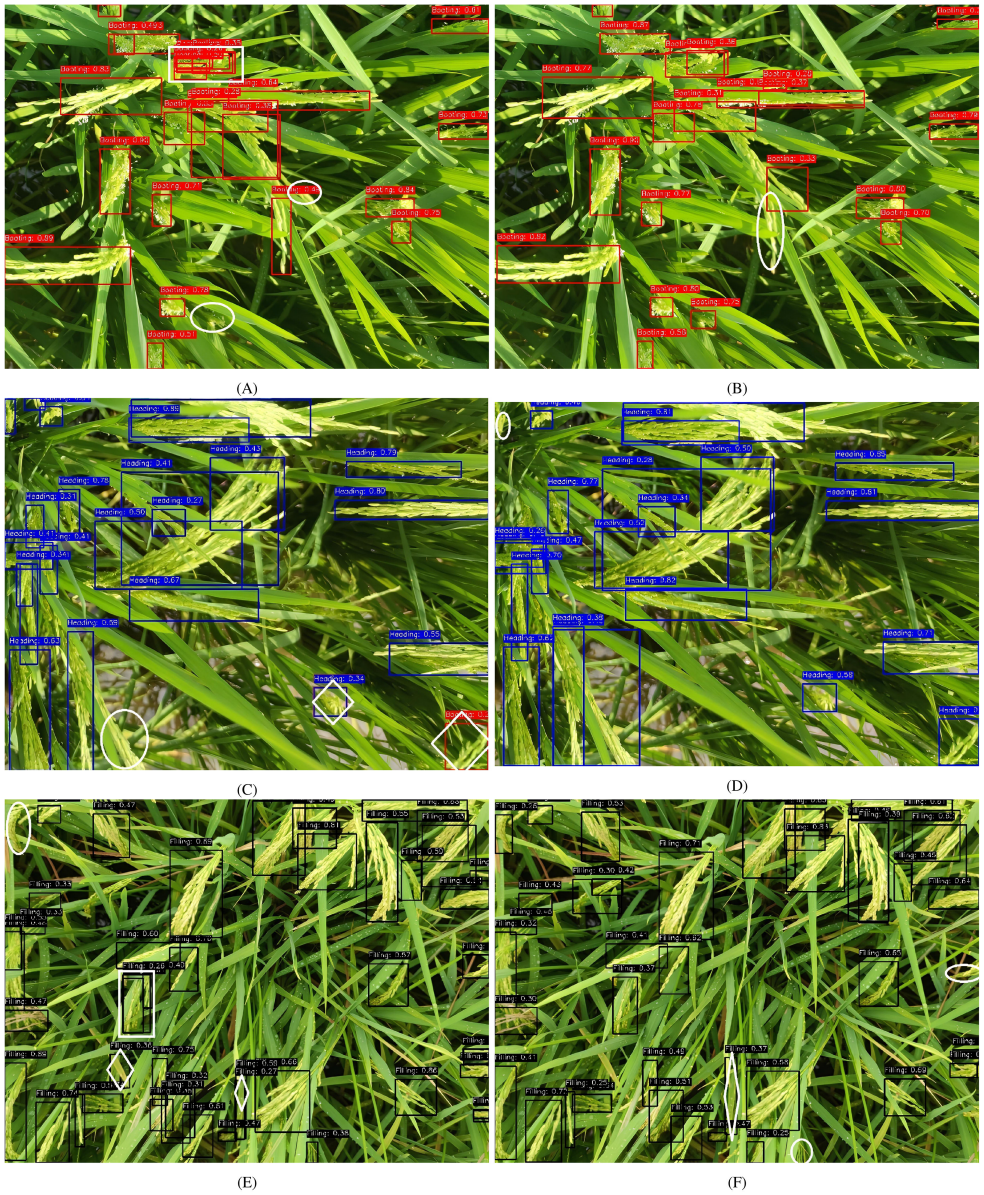
Comparison of the detection results of YOLOv8n (the first column) and YOLO_ECO (the second column). Rice panicles are in the booting stage **(A, B)**, heading stage **(C, D)**, and filling stage **(E, F)**. The red, black, and blue rectangles refer to rice panicle detected as booting, heading, and filling stage, respectively. White circles, hexagons, and rectangles have been manually marked, indicating the missed, false, and duplicate detected rice panicles, respectively.

**Figure 11** shows the comparison detection results between the two architectures. During booting stage observations, YOLOv8n produced two missed and two duplicate detections versus a single missed detection by our model. In particular, YOLO_ECO can accurately detect two occluded panicles and small rice panicles occluded by leaves (the white rectangle and circle in **Figure 11A**). In the heading stage, the YOLOv8n model incorrectly recognized two heading-stage rice panicles as the filling stage and failed to detect one rice panicle. The proposed model correctly detected all the heading stage rice panicles and only one small rice panicle was undetected by it (**Figure 11D**). In the filling stage, as the rice grows, the phenotype of rice becomes complicated, causing serious occlusion scenarios. YOLOv8n cannot accurately detect panicles partially occluded by leaves, causing duplicate detection (the white rectangle in **Figure 11E**). Two leaves were also falsely detected as panicles by YOLOv8n (the white hexagons in **Figure 11E**). In addition, one panicle was not detected by YOLOv8n. However, our model falsely detected one leaf as a rice panicle and had two missed detections. Experimental results confirm YOLO_ECO’s superior detection accuracy and compact architecture on mobile devices, particularly for overlapping and small panicles. The enhanced performance stems from improved feature extraction.

#### 3.4.2 Comparison of different quantization models and input image sizes

As shown in **Table 5**, the quantization model had a more significant impact on model size than the precision. When YOLOv8n used an input image size of 1280, the model sizes of INT8, FP16, and FP32 were 3.0 MB, 6.1 MB, and 12.2 MB, respectively. Compared with FP32, the model size of FP16 and INT8 were compressed by 50% and 75.4%, respectively. Compared with FP32, the mAP values of FP16 and INT8 were slightly decreased by 2.0% and 2.9%, respectively. Similarly, when only quantized models were considered in the comparison between YOLOv8n and YOLO_ECO, a similar variation trend can be observed as that in **Table 3**.

YOLO_ECO_FP32_1280 achieved AP values of 96.4%, 93.2%, and 81.5% for the booting stage, heading stage, and filling stage, respectively, with an overall mAP of 90.4%. In comparison, YOLOv8n_FP32_1280 achieved AP values of 93.7%, 89.3%, with an overall mAP of 88.1%. This indicates that YOLO_ECO outperformed YOLOv8n in all growth stages, particularly in the booting stage. Additionally, YOLO_ECO_FP32_1280 showed a 3.5% increase in mAP compared to YOLO_ECO_FP32_640 despite having a similar model size. This demonstrates that using higher-resolution input images can significantly improve detection accuracy when computational resources allow.

However, for an input image size of 640, INT8 quantization reduced the mAP of YOLOv8n by only 0.7% compared to FP16 quantization while reducing the model size by 52.2%. For YOLO_ECO, INT8 quantization reduced the mAP by 1.8% compared to FP16, with a model size reduction of 52.7%. This suggests that YOLOv8n is less sensitive to INT8 quantization in terms of accuracy, whereas YOLO_ECO experiences a slightly greater accuracy drop after quantization. This may be attributed to the newly added convolutional operations in YOLO_ECO, which may not be fully compatible with existing quantization tools, requiring further optimization.

#### 3.4.3 Performance evaluation on mainstream Android devices

The performance evaluation of different lightweight models on mainstream Android devices is summarized in **Table 6**. In the experiment, “peak/valley” denotes the highest and lowest FPS observed during a 30-minute run, while “Average Detection Speed” refers to the average time required to detect 100 images. The experimental results demonstrate that both the YOLOv8n and YOLO_ECO models exhibit satisfactory real-time detection capabilities on mobile platforms. On the Xiaomi 11 device, which is equipped with a Snapdragon 888 processor, the YOLOv8n model achieves a peak frame rate of 19.68 FPS, with an average detection time of 71 ms per image. In comparison, the optimized YOLO_ECO model improves efficiency, slightly increasing the peak frame rate to 20.87 FPS while reducing the average detection time to 64 ms. Hardware upgrades further enhance computational performance; on the Xiaomi 14, which is powered by an advanced Snapdragon 8 Gen 3 processor, YOLOv8n reaches a peak frame rate of 22.37 FPS, with an average latency of 59 ms. The YOLO_ECO model achieves a peak frame rate of 23.76 FPS and an average processing time of 53 ms on the same device. The observed valley frame rates (ranging from 6.82 to 9.21 FPS) indicate occasional performance fluctuations during complex scene parsing, which suggests a potential area for future optimization. These quantitative comparisons validate the proposed YOLO_ECO architecture, which maintains superior computational efficiency while preserving detection accuracy, making it particularly suitable for resource-constrained agricultural mobile applications that require continuous field monitoring.

**Table 6:** Performance comparison of YOLOv8n and YOLO_ECO models on different devices.

Handset model	Processor	Model	FPS		Average detection speed (ms/images)
			Peak	Valley	
Xiaomi 11	Qualcomm Snapdragon 888	YOLOv8n	19.68	6.82	71
		YOLO_ECO	20.87	7.34	64
Xiaomi 14	Qualcomm Snapdragon 8 Gen 3	YOLOv8n	22.37	7.93	59
		YOLO_ECO	23.76	9.21	53

### 3.5 Recognition of rice growth stage using YOLO-RPD

**Figure 12:**
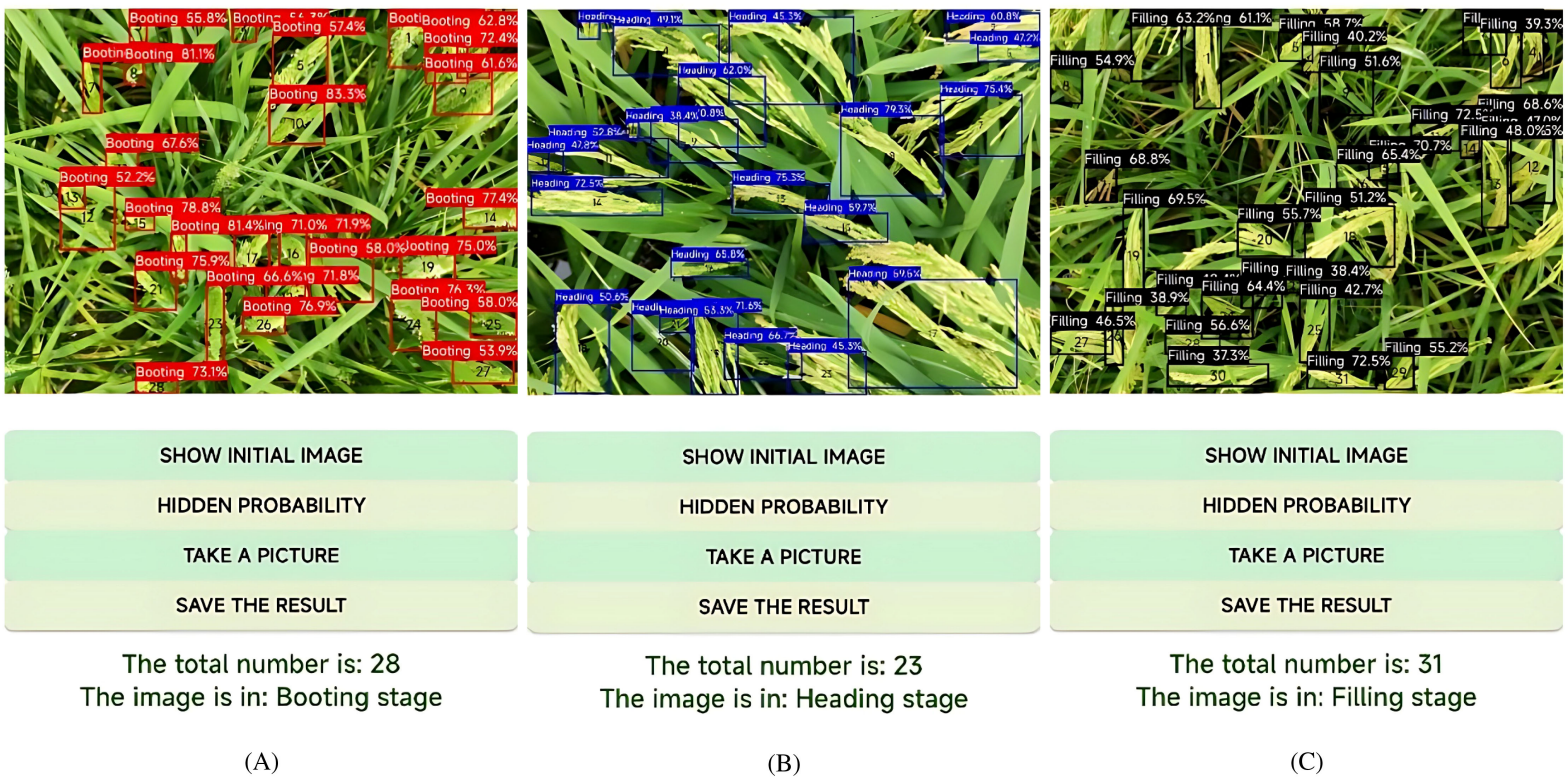
Identification results of rice growth stages using YOLO-RPD: **(A)** booting stage; **(B)** heading stage; **(C)** filling stage.

**Figure 13:**
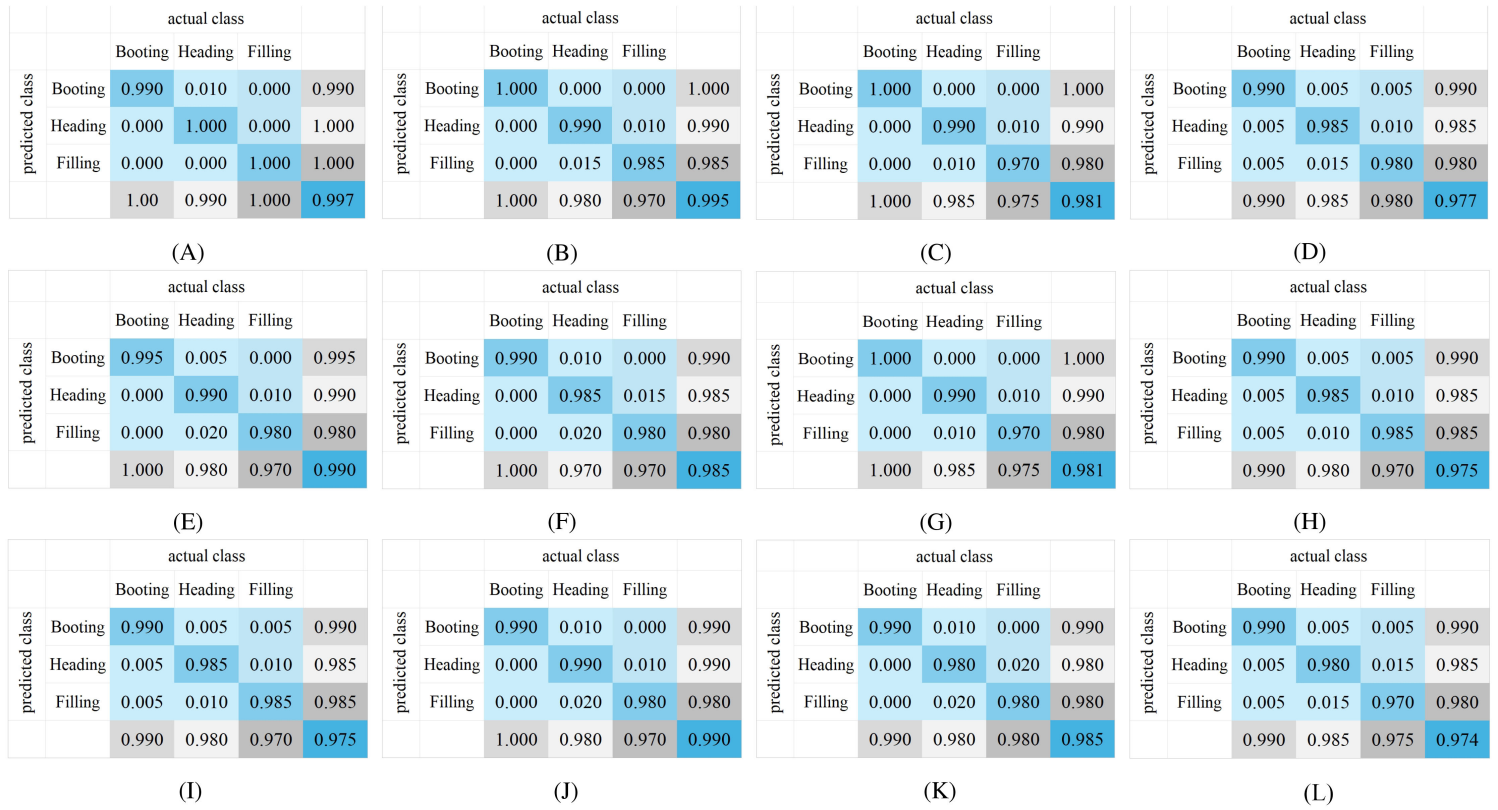
Confusion matrix of the growth stage recognition of different models using YOLO-RPD software: **(A)** YOLO_ECO_INT8_1280; **(B)** YOLOv8n_INT8_1280; **(C)** YOLO_ECO_INT8_640; **(D)** YOLOv8n_INT8_640; **(E)** YOLO_ECO_FP16_1280; **(F)** YOLOv8n_FP16_1280; **(G)** YOLO_ECO_FP16_640; **(H)** YOLOv8n_FP16_640; **(I)** YOLO_ECO_FP32_1280; **(J)** YOLOv8n_FP32_1280; **(K)** YOLO_ECO_FP32_640; **(L)** YOLOv8n_FP32_640.

In addition to rice panicle detection, the YOLO-RPD identifies the growth stage of rice based on the colors of the detection box. Practically, there are significant changes in the external morphological structure of rice panicles in key growth stages, such as shape, color, size, texture, and posture. During the process of image labeling, different colors of annotation boxes were used to identify the growth stages of the rice. Thus, the color of the predicted boxes after model prediction indicated the rice growth stage. As shown in **Figure 12**, after the model prediction, the number of rice panicles in the predicted images was counted according to the color of the detection boxes. The results of rice growth stages were displayed at the bottom of the interface, including the number of different detection boxes, growth stage, and the inference time. **Figure 13A** shows that there were 28 rice panicles detected in the image, all of which were labeled with red boxes indicating the booting stage. Therefore, the rice in the image was recognized as being in the booting stage. **Figure 13B** shows that there was a total of 23 rice panicles detected in the image, among which two were labeled with blue boxes indicating the heading stage. Thus, the rice in the image was identified as being in the heading stage. **Figure 13C** shows that there was a total of 31 rice panicles in the image, all of which were labeled with black boxes indicating the filling stage, therefore, the rice in the image was identified as being in the filling stage.

**Figure 13** shows the confusion matrix results of rice growth stage recognition of different models using YOLO-RPD. Independent test sets were collected in the three growth stages in Exp.1, with 200 images for each growth stage. In **Figure 13**, the first and third columns are the results of the YOLO_ECO models, while the second and fourth columns are the results of the YOLOv8n models. The first and the second columns are results with an input image size of 1,280, while the third and fourth columns are the results with an input image size of 640. The first, second, and third rows are different quantization models of INT8, FP16, and FP32, respectively. YOLO-RPD achieved the best average precision of 99.7% using YOLO_ECO_INT8_1280. Specifically, YOLO_ECO models slightly outperformed the YOLOv8n models. Models with a 1,280 input image size slightly outperformed those with a 640 input image size. The variation of the performance of the models in rice growth stage recognition was similar to that of the rice panicle detection. However, the quantization models showed little impact on the precision of rice growth stage recognition. According to comprehensive analysis, the YOLO-RPD application showed satisfactory performance in the detection of rice panicle and identification of rice growth stages.

### 3.6 Performance in dense and occluded conditions evaluated using heatmaps

**Figure 14:**
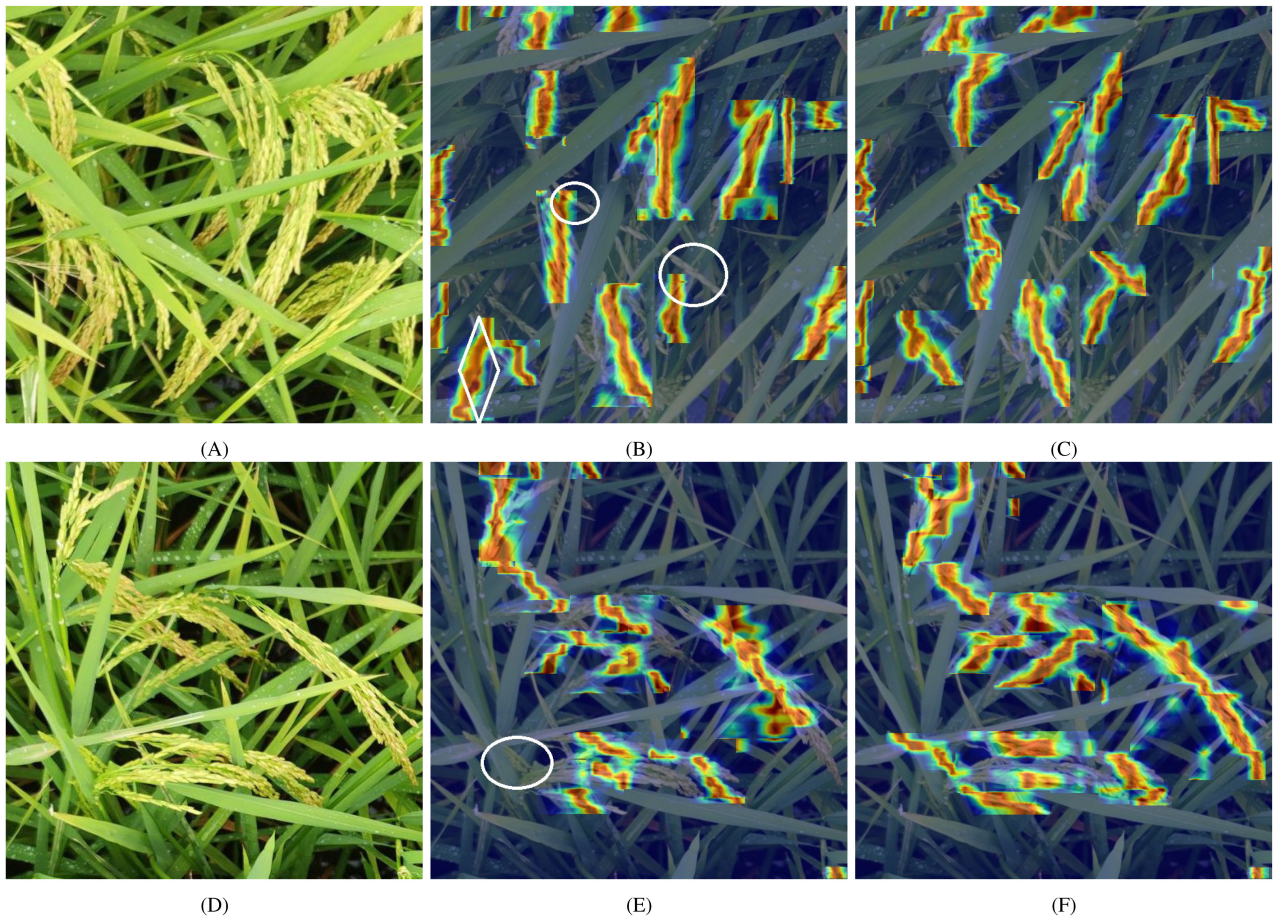
Comparison of the Grad-CAM heatmaps in YOLOv8n and YOLO_ECO: Original images **(A, D)**, heatmaps in YOLOv8n **(B, E)**, heatmaps in YOLOv8n **(C, F)**.

We employed the HiResCAM method to generate interpretive heatmaps to evaluate model performance in dense rice panicle growth conditions and severe occlusion scenarios (Draelos and Carin, 2020). As a class-specific explanation method, HiResCAM ensures exclusive highlighting of the regions actually utilized by the model for its predictions, thereby guaranteeing accurate representation of the model’s attention patterns while avoiding the inherent limitations of traditional methods such as Grad-CAM. The color distribution in these heatmaps visually demonstrates the model’s attention allocation, where red denotes high-attention regions and blue corresponds to low-interest areas. For visual clarity, only heatmaps within the bounding boxes are displayed. **Figure 14** presents a comparative analysis of the heatmaps produced by YOLOv8 and YOLO_ECO, with the first column containing original images, the second column displaying YOLOv8 heatmaps, and the third column presenting YOLO_ECO visualizations. In these visualizations, white circles identify missed detections, while white diamonds indicate false positives. In particular, **Figures 14A, D** demonstrate challenging cases of overlapping rice panicles in high-density arrangements, where distinct identification becomes difficult. Notably, **Figure 14B** reveals both one missed detection and one false positive in YOLOv8n’s output, whereas **Figure 14E** shows an additional missed detection. A comparative analysis of the heatmaps clearly indicates that YOLO_ECO exhibits substantially deeper attention to rice panicles compared with YOLOv8n, suggesting an enhanced feature network focus on critical regions. The fainter areas in YOLOv8n’s heatmaps reflect its diminished recognition capability under occlusion conditions, whereas YOLO_ECO demonstrates superior feature extraction performance.

### 3.7 Comparative analysis in the relevant studies of rice panicle detection

Based on the existing literature, our proposed YOLO-ECO model demonstrates a balanced trade-off between detection accuracy, model complexity, and real-world deployment feasibility. A comparison of different models for rice panicle detection and growth stage recognition is shown in **Table 7**. Compared to Cai et al. (2024), who utilized a MobileNetV3-enhanced YOLOv8 model for rice growth stage recognition, our model achieves a similar level of accuracy (87.2% vs. 84%) while significantly reducing the number of model parameters (1.81M vs. 6.6M), enhancing computational efficiency and deployment feasibility. Additionally, unlike Song et al. (2024), who optimized YOLOv8 for lightweight rice panicle detection with a parameter count of 0.98M but lacked growth stage recognition capability, our model balances both detection tasks while maintaining a compact architecture.

**Table 7:** Comparison of different models for rice panicle detection and growth stage recognition.

Previous studies	mAP (%)	Params (M)	Recognition of growth stage	Deep learning models	Android application
Wang et al. (2022)	92.77	–	No	YOLOv5	No
Tan et al. (2023)	93.7	–	Yes	YOLOv5	No
Qiu et al. (2024)	90.3	7.1	Yes	YOLOv5	No
Song et al. (2024)	95.9	0.98	No	YOLOv8	No
Sun et al. (2024)	86.9	–	No	YOLOv5	Yes
Cai et al. (2024)	84.0	6.6	Yes	YOLOv8	Yes
Ours	87.2	1.81	Yes	YOLOv8	Yes

Compared to YOLOv5-based approaches, Sun et al. (2024) and Wang et al. (2022) achieved high accuracy but focused solely on panicle detection without growth stage classification. Tan et al. (2023) and Qiu et al. (2024) integrated growth stage recognition, yet their models either lack parameter efficiency or deployment considerations. Notably, our YOLO-ECO model, similar to Cai et al. (2024) and Sun et al. (2024), supports Android application deployment, enabling real-time monitoring for practical agricultural applications.

In summary, YOLO-ECO outperforms existing methods in terms of lightweight design, deployment flexibility, and balanced detection capabilities. It offers an effective solution for real-world rice panicle monitoring while ensuring computational efficiency and accuracy in both panicle detection and growth stage recognition.

### 3.8 Discussion, limitations, and future works on real-time rice yield estimation

This study is the first of its kind to provide a smartphone Android application (YOLO-RPD) that can detect rice panicles and identify the rice growth stage in rice images offline or in those acquired online. Moreover, YOLO-RPD offers a range of models that users can choose according to the application requirements. In addition, the study presented an in-depth analysis of the impact of different backbone networks, quantitative models, and input image sizes on the rice panicle detection and growth stage recognition performance. Deep learning models tend to have larger and higher arithmetic power requirements. The experiment results demonstrate that smaller models often have better utility for mobile platforms with limited performance.

For future works, we plan to collect rice images from more diverse scenes, perspectives, and time periods, and integrate multi-source data to enhance the model’s robustness in rice panicle detection. This will make the tool a more viable option for rice phenotype applications. Specifically, we will explore more advanced deep learning algorithms, particularly focusing on advanced backbone networks, to optimize the balance between detection accuracy and time efficiency. We also aim to develop additional functionalities, such as rice yield estimation based on panicle count. Moreover, we plan to implement a real-time video processing function for automated yield estimation, which will be a significant area for future research. As smartphone performance continues to increase, we anticipate that deploying these models on devices with better hardware will further improve detection speed and overall performance, thus enabling more efficient real-time applications.

## 4 Conclusion

Rice panicle detection and growth stage recognition are critical for rice cultivation, and developing deep learning models for mobile phones to address these challenges holds great significance. This study introduces YOLO-RPD, an Android application designed for rice phenotype detection, leveraging an improved YOLOv8 model, YOLO_ECO. The key innovations in YOLO_ECO include replacing the original C2f module with C2f-Faster-EMA in the backbone, simplifying neck complexity with SlimNeck, and enhancing efficiency through the LSCD head. The experimental results demonstrate that YOLO_ECO outperforms traditional models with average precision values of 96.4%, 93.2%, and 81.5% in the booting, heading, and filling stages, respectively. YOLO_ECO also excels in detecting occluded and small rice panicles while reducing model size, computational demand, and parameter count. In terms of model optimization, YOLO_ECO_INT8_640 is the optimal choice when prioritizing model size, achieving an average precision of 83.9% for rice panicle detection and 97.0% for rice growth stage recognition with a model size of just 1.7 MB. For better overall accuracy, YOLO_ECO_FP32_1280 is the best model, with an average precision of 90.4% for rice panicle detection and 99.7% for rice growth stage recognition, though it has a larger model size of 7.5 MB. Looking forward, future work will focus on exploring more advanced deep learning algorithms to optimize the trade-off between detection accuracy and computational cost, as well as expanding the application’s functionality, including real-time video counting and rice yield prediction.

## Data Availability

The original contributions presented in the study are included in the article/supplementary material. Further inquiries can be directed to the corresponding author.
